# Infrared light sensors permit rapid recording of wingbeat frequency and bioacoustic species identification of mosquitoes

**DOI:** 10.1038/s41598-021-89644-z

**Published:** 2021-05-11

**Authors:** Dongmin Kim, Terry J. DeBriere, Satish Cherukumalli, Gregory S. White, Nathan D. Burkett-Cadena

**Affiliations:** 1 Florida Medical Entomology Laboratory, University of Florida, Vero Beach, FL USA; 2 TrakitNow Inc., Columbia, SC USA; 3 Salt Lake City Mosquito Abatement District, Salt Lake City, UT USA

**Keywords:** Biological techniques, Biotechnology, Diseases

## Abstract

Recognition and classification of mosquitoes is a critical component of vector-borne disease management. Vector surveillance, based on wingbeat frequency and other parameters, is becoming increasingly important in the development of automated identification systems, but inconsistent data quality and results frequently emerge from different techniques and data processing methods which have not been standardized on wingbeat collection of numerous species. We developed a simple method to detect and record mosquito wingbeat by multi-dimensional optical sensors and collected 21,825 wingbeat files from 29 North American mosquito species. In pairwise comparisons, wingbeat frequency of twenty six species overlapped with at least one other species. No significant differences were observed in wingbeat frequencies between and within individuals of *Culex quinquefasciatus* over time. This work demonstrates the potential utility of quantifying mosquito wingbeat frequency by infrared light sensors as a component of an automated mosquito identification system. Due to species overlap, wingbeat frequency will need to integrate with other parameters to accurately delineate species in support of efficient mosquito surveillance, an important component of disease intervention.

## Introduction

Mosquitoes are vectors of causative agents for numerous diseases, including malaria, filariasis, dengue, Zika, chikungunya, and encephalitis, ultimately resulting in more than one million deaths annually^1^ and enormous economic losses through the costs of vaccinations, vector controls, and trade embargoes^2^. The recent Zika virus outbreak in Latin America cost approximately USD 18 billion from 2015 to 2017^3^. A number of control strategies have been developed and deployed to combat vector mosquitoes^4^, however, exotic mosquito-borne pathogens continue to be introduced into new areas and the frequency of epidemics is effectively increasing. Mosquito population data are crucial for assessing the local risk of mosquito-borne disease^5^.

Mosquito surveillance is the identification and enumeration of mosquitoes to determine the species composition, abundance, survival, and presence of disease agents prevalent in a given area and time to justify and implement timely data for risk estimate^6^. Important components of efficient and effective mosquito surveillance for making a scientifically-based decision (e.g., mosquito control, infection, insecticide resistance status) rely on consistent specimen collection and precise vector identification by trained staff. However, insufficient and unreliable surveillance data result from labor-intensive/time-consuming tasks, time lags, incorrect classification, and spatial constraints (e.g., remote areas), which undermine surveillance efforts and the corresponding capacity to detect, anticipate, and respond to vector-borne disease^7^. Therefore, advances in automated mosquito capture and reliable identification could provide critical tools effectively to monitor mosquito populations in real-time; a public health necessity in a state that frequently experiences outbreaks of medically important mosquito-vectored pathogens.

Recognition and classification of animals that use flapping flight^8^, based on wingbeat frequency and other parameters, have become increasingly important in the automated identification of these groups^9–11^ using radar and mathematical models or artificial intelligence (AI)^12–14^. Similarly, radar can be used to differentiate flying mammals (bats) into groups (small, medium, or large) according to wingbeat frequency^15^. Much work has been done for revealing aerodynamic properties and kinematic focusing on insect wingbeats of relatively large size such as locusts, hawkmoths, and dragonflies^16,17^. However, mosquitoes display unusual wing kinematics in comparison with other insect groups^18^. Mosquito flight is characterized by long and narrow wings moving through an atypically small stroke amplitude arc, resulting in extremely high wing loading (body mass/wing surface area), low wingbeat amplitude of approximately 40° (150° in fruit fly), and high wingbeat frequency of approximately up to 1000 beats per second^19,20^. The powered and aerodynamic flight strategy in the mosquito facilitates is used for all general activities (e.g., dispersal, foraging, oviposition, evading predators), as well as acoustic communication^21^. The exceptional feature of mosquito wingbeat has frequently been suggested as a physical signature for species-level identification of mosquitoes^22–24^. To date, however, none of the automated mosquito identification systems using a wingbeat has been ready for real-world deployment.

Numerous factors have limited progress toward an automated mosquito identification system based on wingbeat frequency. Importantly, the vast majority of experimental methods to collect mosquito wingbeats have used an acoustic microphone^25–28^. There are some drawbacks to this method. For example, following the inverse squared law, the distance between a mosquito and the acoustic microphone is negatively correlated with a sound's intensity which is a limiting factor for wingbeat data quality. In the case of using a condenser microphone (extremely sensitive), air current and ambient noise are overamplified^23^. Particularly, recording wingbeat in field conditions is challenging due to background “noise” from natural and man-made sources. Also, the difficulty of obtaining and maintaining a variety of mosquito species to collect representative wingbeat frequencies results in poor sample size and low diversity^23^. Consequently, inadequate sampling results in insufficient power to detect a wingbeat distinction for detailed analysis^29^ and even causes contradictory findings (i.e., extremum). Inconsistent data quality and results frequently emerge from different techniques and data processing methods which have not been standardized by comparison to wingbeat collections of numerous species.

In this study, we describe a simple method to detect and record mosquito wingbeat by multi-dimensional optical sensors capturing a reflection of the transient waveform, which consequently avoids the issue of background acoustic noise. Secondly, we describe a wave file library created from the collection of twenty nine mosquito species of vector and nuisance significance that allowed us to generate a robust dataset from a more diverse suite of mosquito species than that used in previous research efforts. Finally, we include variations in the frequency of wingbeats between and within individuals of a single species to investigate whether a wingbeat frequencies differ among individuals of a species or within an individual over time. This work integrates field and laboratory entomology and engineering, with the future goal of developing an automated mosquito identification system that can increase mosquito surveillance capacity.

## Results

Using our semi-automated device, we were able to produce substantial wingbeat datasets from 29 North American mosquito species (Table 1). In total, 21,825 wingbeat files were recorded for building the wave file library which included important vector and nuisance species. Alternating UV-LED lights at either end of the flight tube, controlled by a timer, successfully elicited back-and-forth flight of mosquitoes, increasing the probability of mosquitoes passing through the sensor array and the overall sample size. However, the phototactic response to UV-LEDs differed between mosquito species. The majority of vector and nuisance mosquito species were positively phototactic (e.g., species of *Aedes* and *Psorophora*), while amphibian biting (*Uranotaenia lowii*) and non-nuisance (*Wyeomyia*) were far less responsive to the UV-LED light source. As such, greater numbers of wingbeat files were recorded for vector species (*Aedes aegypti* n = 1161; *Aedes taeniorhynchus* n = 1196; *Anopheles albimanus* n = 3775; *Anopheles quadrimaculatus* n = 2241; *Culex quinquefasciatus* n = 2738). We also observed mosquito flight activity varied according to natural circadian rhythms. Diurnal mosquito species (*Ae. aegypti* and *Ae. albopictus*) were relatively more attracted to UV-LEDs during daylight hours, while nocturnal (active at night) or crepuscular (active at dawn/dusk) species (e.g., *Anopheles* spp.) were more responsive in natural darkness during the night time.

**Table 1:** Observed wingbeat frequency of 29 North American mosquito species.

Species	Sample size (N)	Valid files (N)	Mean wingbeat frequency (Hz)	SD	SE	Upper 95% mean	Median	Lower 95% mean
*Aedes aegypti*	509	1161	498.08	34.19	1.00	500.05	498.05	496.11
*Aedes albopictus*	591	840	536.16	31.24	1.08	538.27	537.11	534.04
*Aedes dorsalis*	40	88	373.92	42.87	4.57	383.01	371.09	364.84
*Aedes infirmatus*	33	162	380.83	29.89	2.35	385.47	378.42	376.19
*Aedes japonicus*	322	690	383.00	37.24	1.42	385.79	375.98	380.22
*Aedes sierrensis*	40	300	425.31	31.55	1.82	428.89	424.81	421.72
*Aedes taeniorhynchus*	335	1196	447.61	40.05	1.16	449.88	444.34	445.33
*Aedes triseriatus*	355	673	395.12	33.41	1.29	397.65	390.63	392.59
*Aedes vexans*	21	714	377.62	26.11	0.98	379.54	371.09	375.71
*Anopheles albimanus*	432	3775	460.02	32.44	0.53	461.06	458.98	458.99
*Anopheles crucians*	107	342	411.71	42.57	2.30	416.24	405.27	407.18
*Anopheles quadrimaculatus*	368	2241	504.52	121.77	2.57	509.56	507.81	499.47
*Culex coronator*	204	813	393.14	38.30	1.34	395.78	385.74	390.50
*Culex interrogator*	32	255	441.56	41.21	2.58	446.64	439.45	436.48
*Culex iolambdis*	51	304	469.14	64.61	3.71	476.43	478.52	461.84
*Culex nigripalpus*	214	720	397.03	38.17	1.42	399.83	390.63	394.24
*Culex pipiens*	568	867	408.39	41.40	1.41	411.15	405.27	405.63
*Culex quinquefasciatus*	798	2738	456.23	30.70	0.59	457.38	454.10	455.08
*Culex restuans*	196	251	341.87	29.19	1.84	345.50	336.91	338.25
*Culex tarsalis*	31	604	395.33	112.06	4.56	404.28	336.91	386.38
*Culiseta incidens*	15	23	426.29	91.70	19.12	465.94	444.34	386.64
*Deinocerites cancer*	79	355	427.65	27.32	1.45	430.50	429.69	424.80
*Mansonia titillans*	4	10	395.02	39.30	12.43	423.13	380.86	366.91
*Psorophora columbiae*	233	721	411.37	32.74	1.22	413.76	405.27	408.97
*Toxorhynchites rutilus*	5	60	600.18	81.81	10.56	621.31	581.06	579.05
*Uranotaenia lowii*	53	369	736.87	58.52	3.05	742.86	742.19	730.88
*Wyeomyia mitchellii*	222	749	438.37	48.60	1.78	441.86	429.69	434.89
*Wyeomyia smithii*	46	53	525.59	173.14	23.78	573.32	444.34	477.87
*Wyeomyia vanduzeei*	240	751	454.91	40.99	1.50	457.84	454.10	451.97

**Figure 1:**
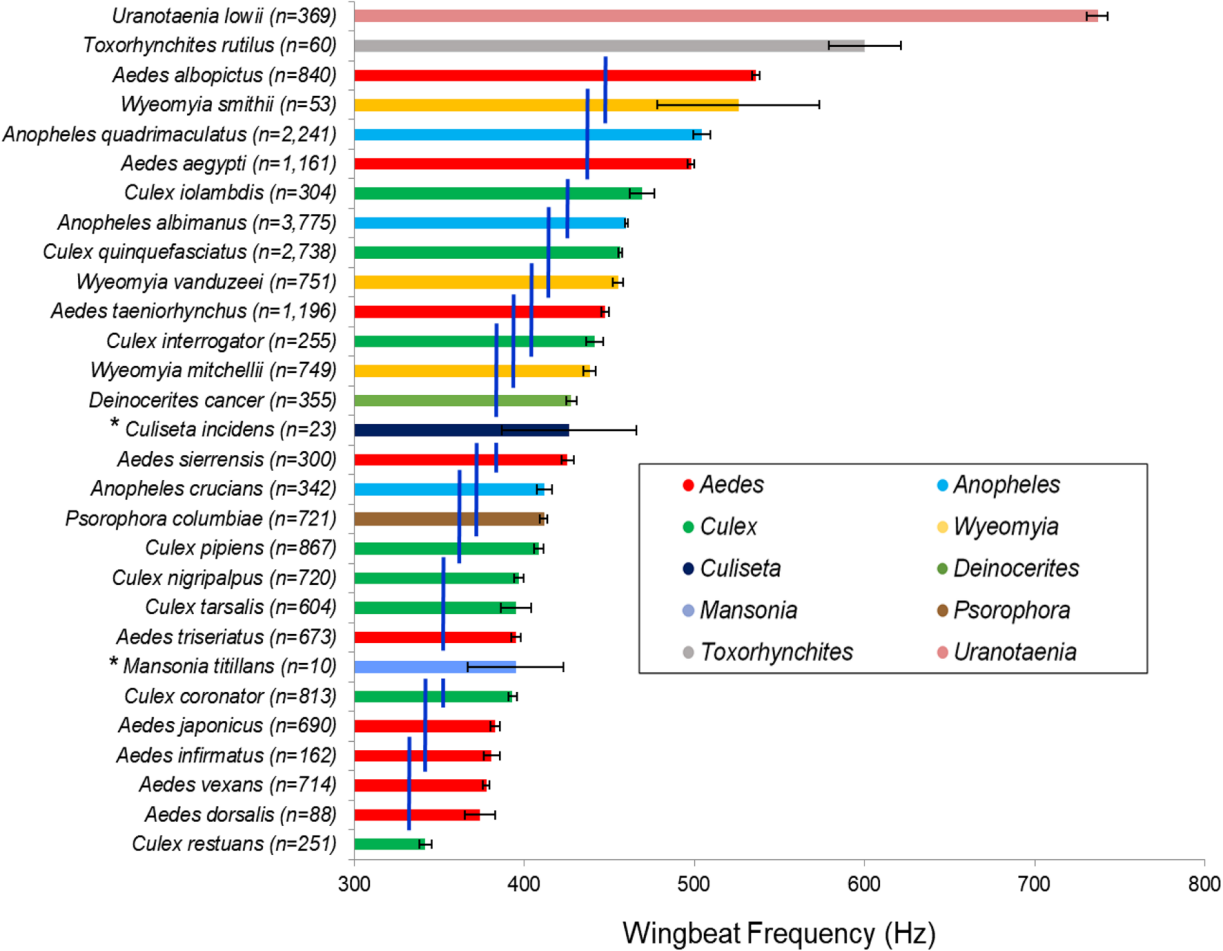
Wingbeat frequencies for 29 mosquito species. Bars (mean wingbeat frequency) are color-coded by the mosquito genus. Error bars represent 95% confidence interval. Overlapping vertical blue lines indicate species means that are not significantly different (*P* > 0.05). *Species with fewer than 20 valid data points (wingbeat files) were excluded from the analysis.

The mean wingbeat frequencies of mosquitoes assayed here spanned 395 Hz (Table 1, Fig. 1), with the lowest frequency observed in *Culex restuans* (341.87 Hz) and the highest observed in *Ur. lowii* (736.87 Hz) (Table 1). Significant differences (F_29_ = 789.1668; *P* ≤ 0.0001) were observed in wingbeat frequencies of mosquito species examined. In pairwise comparisons (Fig. 1), the wingbeat frequency of twenty six species overlapped with at least one other species (not significantly different). Only three mosquito species, *Ur. lowii, Toxorhynchites rutilus,* and *Cx. restuans*, were significantly different (*P* ≤ 0.0001) from all other species, in pairwise comparisons (Fig. 1). Interestingly, all three of these species were at the extremes of the frequency spectrum (Fig. 1). Other species overlapped with one (n = 2), two (n = 5), three (n = 5), four (n = 3), five (n = 4), six (n = 3) or seven (n = 2) species. For example, *Ae. albopictus* was significantly different from 28 species but not significantly different from *Wyeomyia smithii*. The result suggested that it might be feasible to differentiate the wingbeat of *Ae. aegypti* from 27 sympatric species but not *An. quadrimaculatus* or *Wy. smithii*. Within the genus *Aedes*, *Ae. aegypti*, *Ae. albopictus*, *Ae. sierrensis*, and *Ae. taeniorhynchus,* the wingbeat frequencies were all significantly different from one another species (*P* ≤ 0.0001) (Fig. 1). Within the genus *Culex*, *Cx. interrogator*, *Cx. iolambdis, Cx. pipiens, Cx. quinquefasciatus,* and *Cx. restuans*, the wingbeat frequencies were significantly different from one another (*P* ≤ 0.0001) (Fig. 1). Species, within *Anopheles* and *Wyeomyia* genus, the wingbeat frequencies were significantly different from each other (*P* ≤ 0.0001) (Fig. 1).

**Figure 2:**
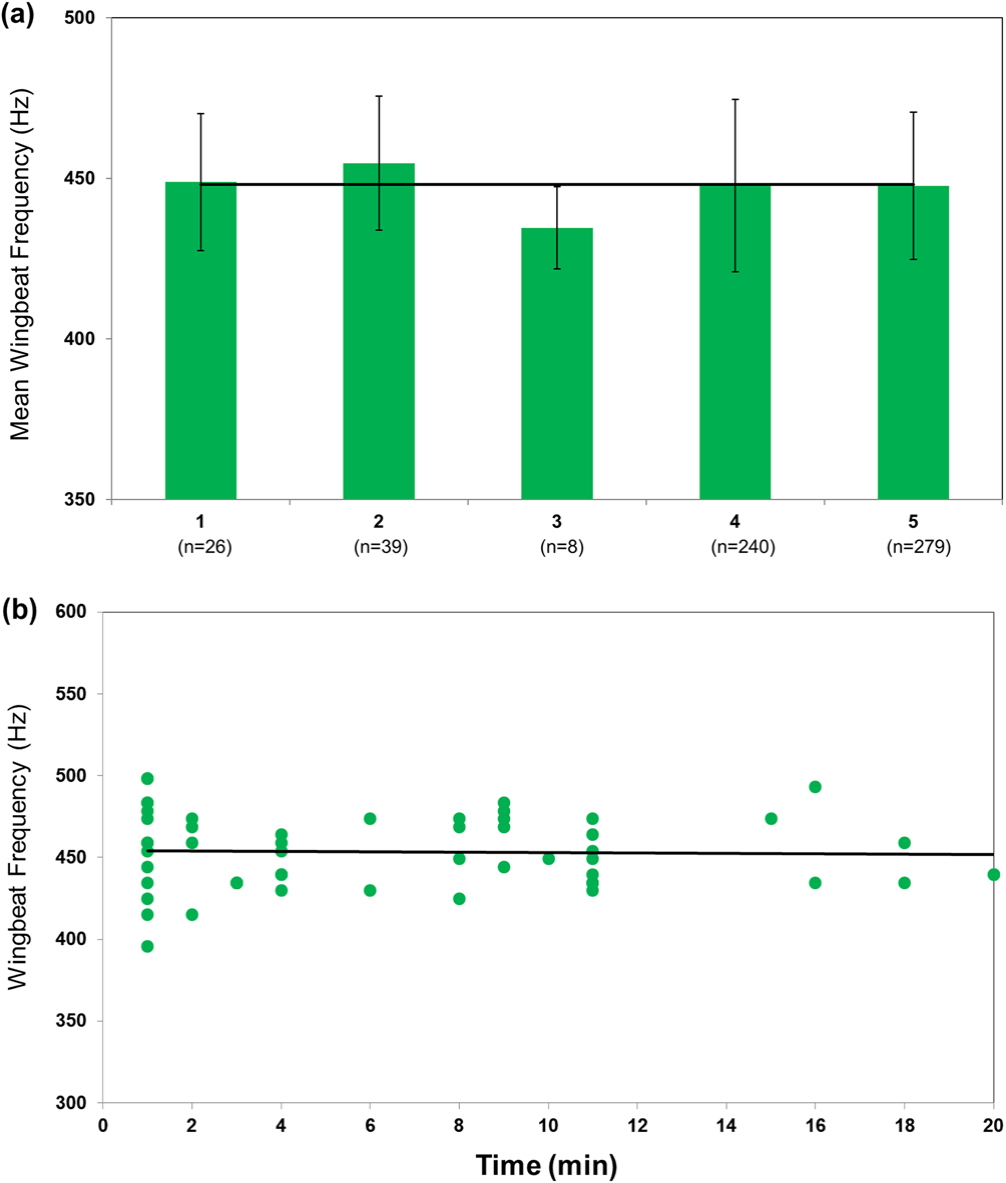
Wingbeat frequencies of *Culex quinquefasciatus*. (**a**) Mean (± standard deviation) wingbeat frequencies of five individuals. Wingbeat files with fewer than five valid data points were excluded from the analysis. (**b**) Changes in wingbeat frequencies over time. Wingbeat frequency was measured using paired infrared emitters and receivers, capturing wingbeat as a function of infrared light interruption.

No significant differences (F_4_ = 1.3788; *P* ≤ 0.2398) were observed in wingbeat frequencies between individual females of *Cx. quinquefasciatus* (Fig. 2). The mean (standard deviation) wingbeat frequencies of five individuals were 448.8 Hz (21.3), 454.7 Hz (20.8), 434.6 Hz (12.8), 447.8 Hz (26.9), and 447.7 Hz (22.9) (Table 2). Also, wingbeat frequencies of female individuals did not differ significantly over time (*r*^2^ = 0.001; *P* = 0.8303) (Fig. 2). Wingbeat frequencies were relatively steady over a period lasting 20 min. The mean wingbeat frequency of mosquitoes was 453.3 Hz accounted for 22.95% of the wingbeat variation.

**Table 2:** Observed Individual wingbeat frequencies of *Culex quinquefasciatus.*

Individual (#)	Valid files (N)	Mean wingbeat frequency (Hz)	SD	SE	Upper 95% mean	Median	Lower 95% mean
1	26	448.84	21.30	4.77	458.22	449.22	439.47
2	39	454.73	20.84	3.90	462.38	449.22	447.07
3	8	434.57	12.79	8.60	451.47	434.57	417.67
4	240	447.77	26.86	1.57	450.86	449.22	444.69
5	279	447.68	22.93	1.46	450.54	444.34	444.82

## Discussion

Our finding that most mosquito species (26/29) overlapped with at least one other species in the distribution of wingbeat frequency has important implications for using this metric as a predictive variable for discriminating mosquito species using an automated identification system. Other variables or parameters will need to be incorporated into the field capture data to produce a robust classification of mosquito species. Potential variables include mosquito size, UV-fluorescence, location (distribution), habitat, and hour of activity. Mosquito species compositions and abundance are known to change along spatial gradients including land cover, altitude, and human/livestock population density^30^. Therefore, detailed information on habitat associations and geographic distributions of individual species could be used to guide algorithms for classifying captured wingbeats to particular species. For example, two species, *Ae. aegypti* and *Wy. smithii* are among 80 mosquito species currently known to occur in Florida^31^ that did not significantly differ in wingbeat frequency (Fig. 1), yet these two species differ in habitat association and geographic distribution. *Aedes aegypti* is considered a domestic (urban/periurban) species that is common in south Florida but rare in northern Florida. *Wyeomyia smithii*, in contrast, is restricted to pitcher plant bogs and is not found in southern Florida. Therefore, a mosquito wingbeat captured in southern Florida is far more likely to be an *Ae. aegypti* than *Wy. smithii.*

Extensive research showed that mosquitoes have specific circadian rhythms (hours of activity), which are dependent on changes in ambient light intensity^32^. Data on circadian rhythms of individual species could constitute an important variable to incorporate into algorithms for classifying captured wingbeats to a particular species. As described above, diurnal mosquito species (*Ae. aegypti* and *Ae. albopictus*) are more active during daylight hours, while many other species are nocturnal or crepuscular^33^. For example, wingbeat frequencies of *Ae. triseriatus* or *Cx. tarsalis* overlapped (not significantly different) and therefore could be misclassified based upon wingbeat frequency alone. However, a mosquito collected at 14:00 h, is much more likely to be *Ae. triseriatus* (a diurnal species) than *Cx. tarsalis*, which bites during crepuscular periods and at night. Abiotic variables, such as ambient temperature, likely impact wingbeat frequency and should be used to modulate algorithms. From our experimental results, for example, *Ae. aegypti* had a mean wingbeat frequency of 498.1 Hz while a previous study recording by a sensitive microphone had a 664.0 Hz (28.6% higher)^34^. A possible explanation for this variability could have resulted from different recording devices or mosquitoes modulating their flight tone for atmospheric conditions such as temperature. For example, wingbeats in the study^34^ were measured at 32.6 ± 0.5 °C while ours were conducted at 23.0 ± 0.5 °C. These differences will be important for compensating wingbeat value in the case of a changing temperature which could be automatically adjustable in a range of wingbeat frequency collected from the future system to identify mosquito species. Species variation in trapping outcome based on trap type would also be an additional predictive variable parameterizing the automated identification system. Mosquito trapping methods vary in their efficacy to capture specific species and life stages which may cause species-specific trap bias^35,36^. For example, the dominant species attracted with light-baited traps may differ from the BioGents Sentinel trap (BG) in a given area where both species co-exist. Our result showed that wingbeat frequencies of *Ae. aegypti* were not significantly different from that of the *An. quadrimaculatus* mosquitoes. However, a mosquito collected from BG trap during daylight hours is far more likely to be *Ae. aegypti* than *An. quadrimaculatus*, which is more responsive to light traps including the New Jersey light trap or the Centers of Disease Control and Prevention (CDC) light trap^37^. In a field deployment, mosquito wingbeat frequencies may incorporate mosquito trap type to increase accuracy and reliability.

Surprisingly, wingbeat did not vary between individuals of *Cx. quinquefasciatus* and even individual mosquitoes exhibited at relatively stable wingbeat frequencies over time (Fig. 2). This low dispersion of wingbeat frequencies found between individuals transitorily could have resulted from laboratory colony being relatively more homogeneous with respect to size comparing to wild strains. However, size variations in both mass and linear dimensions (individual length, width, height) occur in mosquito populations in nature. Physical characteristics including individual mass, wing area, and wingspan are known to impact wingbeat frequencies in flight^38^. For example, flight-associated morphometrics across insect orders suggest that larger insects may not afford lower wing loadings for their body mass, consequently, leading to increased wingbeat frequency comparing to the group with a similar wing length^39^. Also, individual variation in wing length and area is a function of numerous allometric traits including life history (e.g., the availability of nutritional resources during the larval stage), genetics, and physiology^40–42^. For example, the wing length from a wild population of *Cx. quinquefasciatus* females ranging from 2.81 to 4.25 mm was found^43,44^. It is also possible that the wingbeat may be related to wing morphometrics, such as a total wing area as well as the unidimensional wing length^39^. The relationship between wing size and wingbeat frequency is supported by the aerodynamic theory that smaller wings produce less force per beat than larger wings; and therefore, more beats are needed per unit time^39^. This trade-off is likely to explain how energetic costs and pressure change mosquito morphometrics which leads to wingbeat frequency change. As we didn’t cover individual variation in physical appearance (wing length and area) and mass, it is possible that if we manipulate female size by abiotic and biotic conditions, then we may observe a clear relationship between the small and large cohorts in single species. This information would have significant implications for understanding evolutionary trade-off and biomechanical aspects, and for the automated identification system based on a wingbeat may incorporate specific external morphological traits (i.e., wing size and length) to increase accuracy and reliability. An image sensor can be relatively easily added to the sensor array so that insect size can be estimated and used as an additional variable in algorithms to predict mosquito species.

Our novel device permitted rapid measurement of wingbeat frequency data of a large diversity of mosquito species from laboratory colonies and wild strains. Wingbeat frequencies did not cluster phylogenetically (by genus) but were distributed across higher-order taxa. Few species were significantly different than all others examined but wingbeat frequencies of the same species, *Cx. quinquefasciatus*, were not highly variable between and within individuals. However, there are many outstanding questions. For example, variation in the wingbeat frequency from all physiological states including gravid and blood-fed females as well as sexual dimorphism must be resolved about the female wingbeat for parameterizing the automated identification system to successfully increase the sensitivity (number) and specificity (species) for species identification because overlapped wingbeat frequencies preclude the use of species classification alone as a method. Finally, our simple and reliable method opens the way for bringing an automated mosquito identification system into the real world and exploring mosquito flight behavior, which is a key component in biodiversity, ecology, and pathogen dynamics.

## Materials and methods

### Instrument to capture wingbeat frequency

**Figure 3:**
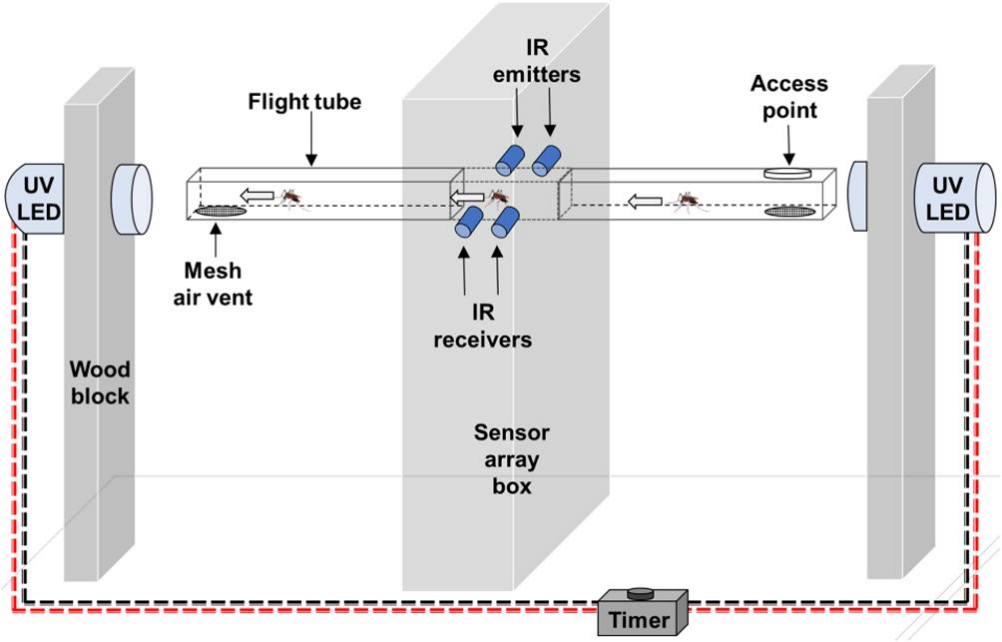
Device for recording mosquito wingbeats. Female mosquitoes pass between IR emitters and receivers, attracted back and forth through the flight tube by alternating UV LED flashlights, controlled by a timer.

The novel wingbeat recording device included a sensor array with two pairs of infrared emitters (Lite-on, Inc., Taipei, Taiwan) and corresponding receivers (Lite-on, Inc., Taipei, Taiwan) covering horizontal and vertical planes, which captured wing light disturbance when mosquitoes flew through the array (Fig. 3). The sensor array also contained a cellular modem (Simcom, Shanghai, China), a secure digital card (SanDisk, CA, USA), with supporting electronics powered by a 12 V DC source (Fig. 3) provided by TrakitNow, Inc., SC, USA. A mosquito flight apparatus, passing through the sensor array, was made with a section of 18.0 cm × 1.5 cm × 1.5 cm transparent polypropylene tube (ClearBags, CA, USA). The tube was close-ended and had a 1.5 cm diameter of hole as an access point on the top for mosquito release and withdrawal. Sections of the flight tube within the sensor array were covered with 5.0 cm × 2.0 cm × 1.0 cm of Near-Infra-Red filters (Astra Products, Inc., NY, USA); eliminating unwanted visible spectrum. A pair of 1.5 V ultra-violet light-emitting diode (UV-LED) bulbs in parallel connection was positioned at both ends of the flight apparatus that was operated from 6.0 V by DC adaptor. Voltage was applied to the infrared emitters to maintain a 1.5 vdc ± 50 mv output from the receivers prior to sensing a rapid drop in voltage of at least 100 mv and 200 ms following the sensing of the rapid drop indicating a wingbeat, using an STM32F540G microcontroller. An ESP32-Room microcontroller (Espressif Systems, Shanghai, China) was added to control the on/off interval of the UV-LED such that the UV-LEDs alternated on and off in order to attract mosquitoes flying back and forth through the flight tube and sensor array, continually capturing wingbeats.

Sample size varied from availability for mosquito species (Table 1). Maximums for sample size were twenty mated female mosquitoes (3–5 d old) that had never received a blood meal, were collected using an aspirator were released into the flight apparatus. It allowed acclimating to the environment for 5 min. Constant environmental conditions (23.0 ± 0.5 °C, 60.0 ± 5.0% RH) were used. Especially, the wingbeat files from species that medically important (*Ae. aegypti*, *Ae. albopictus*, *An. albimanus*, and *Cx. quinquefasciatus*) were collected with a minimum of 2000 valid files. When a mosquito passed through the sensor array, the mosquito movement partially occluded the infrared light, resulting in tiny light disturbances (fluctuations). Wingbeat was captured by infrared receivers intermediate in the opposite site as changes in currents which were connected to an electronic board to filter and amplify the wingbeat signals; sensed by the triggering of the STM32’s a rapid drop off in voltage as sensed by the microcontroller and ended after a 200 ms recording interval. The voltage was adjusted by the STM32 after the interval (the receivers’ output was more than 50 mv out of calibration). The data during the 200 ms interval was recorded, stored, and sent to AWS via a SIMCOMM 5320 cellular modem programmed by Trakitnow, Inc. In order to determine whether the mosquito wingbeat variation was consistent between and within an individual in the same species, individual mosquito wave files in *Cx. quinquefasciatus* species were collected. We introduced an individual mosquito into the flight apparatus with the sensor array in succession and was removed over valid data points (minimums for sample size). The experiment was replicated five times.

### Mosquito samples

A wingbeat library was constructed with mosquitoes from laboratory colonies in Florida and Utah and supplemented with field collections of species not colonized. Mosquitoes from colonies included three *Aedes* spp. (*Ae. aegypti* (L.)*, Ae. albopictus* Skuse*,* and *Ae. sierrensis*), two *Anopheles* spp. (*An. albimanus* and *An. quadrimaculatus*), two *Culex* spp. (*Cx. quinquefasciatus* and *Cx. pipiens*), *Tx. rutilus,* and *Ur. lowii*. Other nuisance and vector mosquito species were captured from light traps as adults or reared to adulthood from larval forms/egg rafts. These included *Culex* spp. (*Cx. nigripalpus, Cx. coronator, Cx. tarsalis, Cx. iolambdis, Cx. interrogator,* and *Cx. restuans*), *Aedes* spp. (*Ae. dorsalis, Ae. taeniorhynchus, Ae. vexans, Ae. infirmatus, Ae. triseriatus*, and *Ae. japonicas*), *An. crucians*, *Wyeomyia* spp. (*Wy. smithii*, *Wy. vanduzeei*, and *Wy. mitchellii*), *Psorophora columbiae*, *Culiseta incidens*, *Deinocerites cancer*, and *Mansonia titillans* (Table 3). Laboratory colony or wild strains of species were maintained in an environmental chamber (27.0 ± 0.5 °C, 80.0 ± 5.0% RH, and 14:10 (L:D) h photo regime) at the Florida Medical Entomology Laboratory (FMEL) at University of Florida, Vero Beach, Florida, USA. In general, larvae were reared in 24.8 cm × 19.7 cm × 3.8 cm enamel pan containing 1.0 L of distilled water and fed an equal mixture of brewer's yeast and lactalbumin or diet of fish food, TetraMin (Tetra, Virginia, USA) on a standardized mosquito rearing schedule^45^. Pupae were collected daily and placed in a 30 ml plastic cup at a density of up to 50/cup. Containers were partitioned into groups of three and placed into 24.0 cm × 24.0 cm mesh plastic screen cages (BioQuip Products Inc., California, USA) for adult eclosion. Emergent adults were provided ad libitum with a 10% sucrose solution placed on absorbent cotton and inserted in a 30 ml plastic cup placed inside each adult cage. The adults were anesthetized with carbon dioxide (CO_2_) for 5 min then immediately identified under the dissecting microscope according to standard keys^46^ before use.

**Table 3:** Source and medical importance of twenty nine mosquito species used in wingbeat assays.

Species	Source	Source location	Importance (vector)
*Aedes aegypti*	Lab colony	FL	CHIKV, DENV, MAYV, YFV. ZIKV
*Aedes albopictus*	Lab colony	FL	CHIKV, DENV, YFV, ZIKV
*Aedes dorsalis*	Field	UT	CEV, WEEV
*Aedes infirmatus*	Field	FL	WNV, EEEV
*Aedes japonicus*	Field	NC	WNV, JEV, SLEV
*Aedes sierrensis*	Lab colony	UT	WEEV
*Aedes taeniorhynchus*	Field	FL	
*Aedes triseriatus*	Field	NC	LACV, EEEV, WEEV
*Aedes vexans*	Field	FL	TAHV
*Anopheles albimanus*	Lab colony	FL	Malaria
*Anopheles crucians*	Field	FL	Malaria
*Anopheles quadrimaculatus*	Lab colony	FL	Malaria
*Culex coronator*	Field	FL	WNV
*Culex interrogator*	Field	FL	
*Culex iolambdis*	Field	FL	VEEV
*Culex nigripalpus*	Field	FL	WNV, SLEV
*Culex pipiens*	Lab colony	UT	RVFV, SINV, WNV
*Culex quinquefasciatus*	Lab colony	FL	HLF, WNV, SLEV
*Culex restuans*	Field	FL	WLEV, WNV
*Culex tarsalis*	Field	UT	SLEV, WNV, WEEV
*Culiseta incidens*	Field	CA	
*Deinocerites cancer*	Field	FL	
*Mansonia titillans*	Field	FL	VEEV
*Psorophora columbiae*	Field	FL	VEEV
*Toxorhynchites rutilus*	Lab colony	FL	
*Uranotaenia lowii*	Field	FL	
*Wyeomyia mitchellii*	Field	FL	
*Wyeomyia smithii*	Lab colony	UT	
*Wyeomyia vanduzeei*	Field	FL	

Pathogen associations from Mullen and Durden 2019^47^.

California encephalitis virus (CEV), Chikungunya virus (CHIKV), Dengue fever (DENV), Eastern equine encephalitis virus (EEEV), Human lymphatic filariasis (HLF), Japanese B encephalitis virus (JEV), LaCrosse encephalitis virus (LACV), Mayaro virus (MAYV), Rift Valley fever virus (RVFV), Sindbis virus (SINV), St. Louis encephalitis virus (SLEV), Tahyna virus (TAHV), Venezuelan equine encephalitis virus (VEEV), West Nile virus (WNV), Western equine encephalitis virus (WEEV), Yellow fever virus (YFV), Zika virus (ZIKV).

### Statistical analysis

Mosquito wingbeat data was collected only in frequency spectrum range from 100 to 2000 and selected based on the validated files to reduce the wingbeat error rate. Extracted wing-beat fragments in each mosquito spp. were altered from mbn to wav (Waveform Audio File Format) using Wave Converter with python 3.7 (Python Software Foundation, OR, USA). Fundamental wingbeat frequencies were analyzed by one-way ANOVA followed by Tukey's HSD test for multiple comparisons of means to test the effect of species and individual differences. Alpha was set at 0.05 for all statistical tests.
